# Obesity and Comorbidity: Could Simultaneous Targeting of esRAGE and sRAGE Be the Panacea?

**DOI:** 10.3389/fphys.2019.00787

**Published:** 2019-06-25

**Authors:** Chinedum Eleazu, Norsuhana Omar, Oon Zhi Lim, Boon Seng Yeoh, Nik Hazlina Nik Hussain, Mahaneem Mohamed

**Affiliations:** ^1^Department of Physiology, School of Medical Sciences, Universiti Sains Malaysia, Kubang Kerian, Malaysia; ^2^Department of Chemistry/Biochemistry/Molecular Biology, Alex Ekwueme Federal University, Ndufu-Alike, Ikwo, Nigeria; ^3^Women’s Health Development Unit, Universiti Sains Malaysia, Kubang Kerian, Malaysia

**Keywords:** obesity, nutrition, metabolic dysregulation, receptor for advanced glycation end products, metabolic syndrome

## Abstract

Obesity, a chronic multifaceted disease, predisposes its patients to increased risk of metabolic disorders such as: diabetes mellitus, cardiovascular diseases, dyslipidemia, etc. Recent studies reported it to be amongst the leading causes of deaths in the world. Although several treatment options for obesity abound, many of them have not been able to successfully reverse the existing obesity and metabolic dysregulation. This has therefore warranted the need for either alternative therapies or diversification of the treatment approach for obesity and its comorbidity. When the receptor for advanced glycation end products (RAGE) interacts with its ligand, RAGE-ligand activates an inflammatory signaling cascade, that leads to the activation of nuclear factor kappa B (NF-κB) and transcription of inflammatory cytokines. This action has been associated with the development of obesity and its mediated metabolic dysregulation. In view of the increasing prevalence of obesity globally and the potential threat it places on life expectancy, this article reviewed the promising potentials of targeting endogenous secretory receptor for advanced glycation end products/soluble receptors for advanced glycation end products signaling as a treatment approach for obesity. We carried out a literature search in several electronic data bases such as: Pubmed, Pubmed Central, Google, Google Scholar, Scopus, and Medline from 1980 to 2019 to acquire the status of information concerning this. The article suggests the need for the development of an esRAGE/sRAGE targeted pharmacotherapy as a treatment approach for obesity and its comorbidity.

## Introduction

Obesity is a chronic metabolic disease that is characterized by excess body fat as a result of hyperplasia and hypertrophy of the adipocytes (Renata et al., 2018; Egedigwe-Ekeleme et al., 2019). Obesity which can be induced by overnutrition and characterized by inflammation and oxidative stress, predisposes its patients to increased risk of diabetes mellitus (T2DM), cardiovascular diseases, dyslipidemia, cancer, etc. (Priyanto et al., 2016; Richard et al., 2019). Furthermore, recent studies reported it to be one of the leading cause of deaths in the world with an annual mortality rate of 2.8–3.4 million (Egedigwe et al., 2016; Priyanto et al., 2016; Victoria et al., 2018).

Although there are many options for the treatment of this disease such as dietary management, exercise, life-style changes, weight-loss medications, and weight-loss surgeries (Nan-Nong et al., 2016), many of them have not been able to successfully reverse obesity and its associated metabolic dysregulation or comorbidity (Burke et al., 2018).

The receptor for advanced glycation end products (RAGE) was reported to be a multi-ligand cell surface protein (Miranda et al., 2018). When bound to its ligand, RAGE initiates an inflammatory signaling cascade, that leads to the activation of nuclear factor kappa B (NF-κB) and transcription of inflammatory cytokines. This action has been associated with the development of obesity and its co-morbidity (Vazzana et al., 2012). Therefore, attenuation of the signaling of RAGE has been suggested as a veritable approach for the treatment of obesity and its comorbidity (Miranda et al., 2018).

The isoforms of the soluble receptors for advanced glycation end products (sRAGE) act as decoy receptors for RAGE by sequestering RAGE ligands and attenuating RAGE signaling. These isoforms include: cleaved RAGE (cRAGE) which is produced through proteolytic shedding of the RAGE and the endogenous secretory RAGE (esRAGE) which is formed by splicing of the pre-RNA of RAGE (Miranda et al., 2018).

Recently, several therapeutic properties have been credited to these sRAGE such as: antidiabetic, anti-inflammatory, and antioxidant properties (Parisa and Ali, 2011; Lorenzi et al., 2014; Miranda et al., 2018) and for which some reviews are available on them in literature. Surprisingly, reviews on the potential usefulness of these decoy receptors as targets for the treatment of obesity are lacking in literature.

Given the increasing prevalence of obesity and its comorbidity globally, the need to diversify its treatment approach has become a necessity. Since attenuation of the signaling of RAGE has been suggested as a beneficial approach for the treatment of obesity and its comorbidity and being that these isoforms of RAGE act as decoy receptors for RAGE, diminishing its signaling (Miranda et al., 2018), the present article reviewed the concept of targeting of esRAGE and sRAGE signaling as a beneficial approach for the treatment of obesity.

## Materials and Methods

We conducted our literature search in several electronic data bases such as: Pubmed, Pubmed Central, Google, Google Scholar, Scopus, and Medline from 1980 to 2019 to obtain the current status of information regarding our concept using keywords such as: obesity, T2DM, advanced glycation end products (AGEs), RAGE, esRAGE, and sRAGE. The findings we got from these data bases are hereby reported in this review.

## Definition of Obesity

Obesity could be defined as a chronic multifaceted disease that is characterized by excess body fat due to hyperplasia and hypertrophy of adipocytes (Renata et al., 2018). It is a condition that has been associated with oxidative stress, inflammation and apoptosis. Obesity arises when energy intake exceeds expenditure (overnutrition), producing toxic substances such as AGEs (Vasudevan et al., 2013; Maria et al., 2016).

## Etiology of Obesity and Prevalence

The etiology of obesity arises from an imbalance between energy intake and expenditure as previously stated, with resultant deposition of fat in the adipose tissue. Several factors have been linked to this imbalance such as: environmental, excess calorie intake with decreased energy expenditure, neurologic, genetic, biochemical, metabolic, and endocrine factors (Ferrier, 2014; Renata et al., 2018; Torres-Villarreal et al., 2018).

The net effect of deposition of fat in the adipose tissue is adipose tissue accumulation which involves an increase in the amount of lipids in the adipocytes (hypertrophy) and the formation of new adipose cells (hyperplasia), otherwise known as adipogenesis (Torres-Villarreal et al., 2018).

According to the reports that were given by WHO in 2016^1^, more than 1.9 billion adults, aged 18 years and above were overweight, out of which 650 million or more were obese. Furthermore, several persons in the world live in countries where overweight and obesity kill more people than being underweight and about 41 million children below 5 years of age were reported to be overweight or obese in 2016 while over 340 million children and adolescents between 5 and 19 years were reported to be overweight or obese in 2016.

The prevalence of overweight and obesity were highest in the WHO Regions of the Americans and lowest in the WHO Region for South East Asia, according to the reports by WHO^1^.

In the WHO Regions for Europe, Eastern Mediterranean and the Americans, over 50% of women were reported to be overweight. In addition, in all of the three regions, roughly half of overweight women were reported to be obese (23% in Europe, 24% in the Eastern Mediterranean, 29% in the Americans, respectively). Further more, in all of the WHO regions that were reported, women were found to be more likely to be obese than men. In the WHO regions for Africa, Eastern Mediterranean and South East Asia, women were also reported to have twice the obesity prevalence of men ^2^.

## Measurement of Obesity

Due to the fact that the amount of body fat is not easy to measure directly, obesity is therefore determined indirectly using the body mass index (BMI) which is measured as the ratio of the body weight (g) to the length (cm^2^) and which has been found to correlate with the amount of body fat in most individuals (Ferrier, 2014).

Previous studies also showed the BMI to be a useful and reliable index of measurement of population-based obesity, and it was also reported to be a simple tool for the classification of underweight and overweight individuals (Ruth and Jean, 2010).

Following the classification of underweight, overweight and obesity by WHO based on BMI cutoffs, an individual with a BMI of <18.5 is considered to be underweight; a BMI of 18.5–24.9 is considered to be in the normal range; a BMI of 25–29.9 is considered to be overweight; a BMI of 30–34.9 is considered to be moderate obesity (class I); a BMI of 35–39.9 is considered to be severe obesity (class II) whereas a BMI ≥ 40 is considered to be very severe or morbid obesity (class III) (World Health Organization [WHO], 2000; Ferrier, 2014).

Measurement of the waist circumference using a tape has also been found to be diagnostic of obesity as it reveals the amount of fat (visceral fat) in the central abdominal region of the body (Ferrier, 2014). This approach has also been reported to reduce the risk of developing cardiovascular diseases, independent of BMI (Ferrier, 2014).

## Metabolic Effects of Obesity

The early metabolic effects of obesity involve a range of metabolic changes such as: oxidative stress, inflammation, markers of metabolic syndrome such as: dyslipidaemia [high levels of triacylglycerols (TAG), low density lipoprotein (LDL) and insulin resistance, and low level of high density lipoprotein (HDL)] (Geoffrey, 2015). Following these early metabolic effects, progression to type 2 diabetes mellitus (T2DM), cardiovascular disease (other markers of metabolic syndrome) and mortality are likely to occur unless there are significant interventions although not all forms of obesity progress to these^3^ (Geoffrey, 2015).

Furthermore, being overweight leads to a 2 or more fold increased risk of dyslipidaemia and metabolic syndrome (Geoffrey, 2015) while it leads to a fourfold increased risk of T2DM (Cameron et al., 2009a,b; Geoffrey, 2015). Other metabolic diseases that have also been associated with obesity include: musculoskeletal disorders (e.g., osteoarthritis), renal impairment and cancer^3^.

Childhood obesity on the other hand, has also been associated with mortality and disability in adulthood. Other metabolic effects of childhood obesity include: difficulty in breathing, increased risk of fractures, hypertension, cardiovascular disease, insulin resistance, and psychological deffects.

## Current Treatment Options for Obesity

The treatment goals for obesity are to either reduce or maintain an ideal body weight for a long period of time or to prevent an additional weight gain for obese individuals who are unable to achieve the desired weight loss (Christopher and Kumar, 2009).

Some of the essential factors that are considered when weight loss therapies are prescribed include: age and sex of the patient, degree of obesity, individual health risks, ability to exercise, psychobehavioral characteristics and results of previous attempts to lose weight (Christopher and Kumar, 2009). The various treatment options for obesity as discussed in this review include: modification of lifestyle (dietary/exercise/behavioral approach), pharmacotherapy and surgery (Snow et al., 2005; Sacks et al., 2009; Ferrier, 2014).

## Modification of Lifestyle

This includes: dietary modification, increased physical activity and behavioral approach to achieve weight loss.

### Dietary Modification

Dietary modification or intervention includes: intake of diets that are low in calories, vegetarian diets, all of which have the overall objective of achieving a weight loss of 500–1,000 kcal/d for obese subjects and 300–500 kcal/day for overweight subjects (Christopher and Kumar, 2009). However, studies have shown that most individuals who used dietary intervention to enhance weight loss had rebound weight gain when the intervention was stopped (Ferrier, 2014).

### Physical Activity

An increase in physical activity can lead to an energy deficit thereby ehnancing considerable weight loss and as such, it is recommended as a key component of programs that are targeted at achieving weight reduction (Ferrier, 2014).

Physical activity includes walking or swimming for about 30–45 min (3–5 days per week) for short term goal; whereas for long term goal, it involves engaging in 30 min of moderate to intense physical activity daily or most days of the week (Christopher and Kumar, 2009). Studies have also shown that physical activity enhances cardiopulmonary fitness and reduces the risk of developing cardiovascular diseases, independent of weight loss (Ferrier, 2014). In addition, combining dietary intervention and physical activity has been shown to be more effective in achieving the desired weight reduction compared to any one of the two methods (Christopher and Kumar, 2009).

### Behavioral Therapy

This approach involves methods that are designed to enhance weight reduction behaviors. It came about from the previous notion that obesity resulted from inadequate adaptive eating and exercise habits, which could be corrected by the application of learning principles although other factors as reported in this review have now been known to contribute to the development of obesity.

This therapy specifies the goals for weight reduction and the strategies to overcome the barriers to achieving them and it includes: self-monitoring, stimulus control, nutrition education, slower eating habbit, and others (Christopher and Kumar, 2009).

## Pharmacotherapy/Drug Therapy

This method comes into play when lifestyle interventions fail to achieve the desired weight reduction and previous studies recommended that this method should be initiated concomitantly with lifestyle modifications (Christopher and Kumar, 2009; Ferrier, 2014; Yoon et al., 2016). Following the recommendation by the Endocrine Society Clinical Practice Guideline for the pharmacological treatment of obesity, this method is to be used only for patients that have a BMI > 30 or have a BMI ≥ 27 but with comorbidity (Apovian et al., 2015; Nan-Nong et al., 2016).

Previously, several drugs were used to treat obesity but some of them were terminated due to the adverse effects that were associated with their usage (Chandrasekaran et al., 2012; Nan-Nong et al., 2016). Currently, orlistat, lorcaserin, phentermine, and phentermine/topiramate are the drugs that are being used for the treatment of obesity as approved by the United States Food and Drug Administration (Dietrich and Horvath, 2012; Weaver, 2018). Their mechanisms of action are described in Table 1. However, despite the successes that were recorded with their usage, some patients that are placed on them revert to increased weight gain when their usage is terminated (Egedigwe-Ekeleme et al., 2019).

**Table 1:** Mechanisms of action of currently used anti-obesity drugs and their side effects.

Drug	Mechanism of action	Side effect	Year approved
^∗^Phentermine	Causes the release of Noradrenaline, serotonin Dopamine, all of which lead to suppression of appetite	Increases blood pressure insomnia, restlessness.	1959
^∗∗^Orlistat	Inhibits pancreatic lipase activity and delays absorption of fat	Steatorrhea, Altered absorption of fat soluble vitamins.	2009
^∗∗^Lorcaserin	Receptor agonist for 5-hydroxytryptamine, decreases appetite enhances satiety	Could lead to liver and kidney Injury, flatulence, headache, nausea, dizziness, disorder of the heart.	2012
^∗∗^Phentermine	Enhances release of dopamine and serotonin, Decreases appetite	Increases heart rate;	2012
^∗∗^+ topiramate	Enhances satiety	Birth defects	

*^∗^Recommendation is for short term use; ^∗∗^ Recommendation is for long term use; Sources: Dietrich and Horvath (2012), Klein and Romijn (2016), Lee et al. (2016), Yoon et al. (2016), Weaver (2018).*

## Surgery

This method is applicable to individuals who are morbidly obese with a BMI ≥ 40 kg/m^2^ or a BMI ≥ 35 kg/m^2^ and significant obesity-related comorbidities. Furthermore, gastric bypass and restriction surgeries are made use of in this approach (Christopher and Kumar, 2009). The approach is quite risky and being that morbidly obese patients have little physiologic reserve, any postoperative complication can be life threatening if not well managed (Christopher and Kumar, 2009). However, this method has been reported to be very effective in achieving weight loss in morbidly obese subjects when it is well managed (Ferrier, 2014).

As was recommended by the Endocrine Society Clinical Practice (Apovian et al., 2015; Nan-Nong et al., 2016), the best modality for the treatment of obesity or achieving a weight loss is a combination of appropriate dietary, lifestyle changes and moderate-intense exercise.

However, studies including both epidemic and clinical studies have also shown that it could be challenging to maintain a consistent lifestyle modification in the long term (Thomas et al., 2014; Nan-Nong et al., 2016).

## Ages-Overview

**FIGURE 1:**
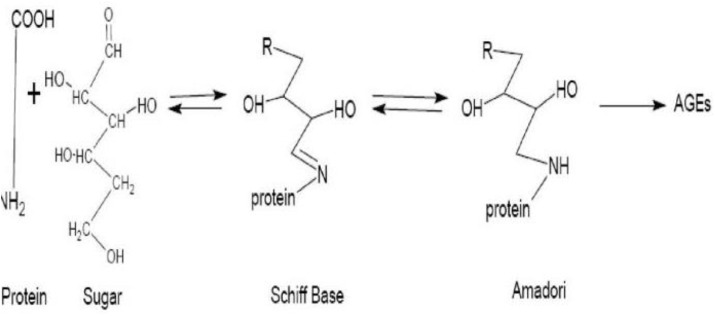
Diagrammatic representation of the formation of advanced glycation end products. Source: Parisa and Ali (2011).

AGEs are compounds that are formed from proteins and peptides by non-enzymatic glycoxidation reactions after interaction with aldose sugars (Chih-Tsueng et al., 2014; Richard et al., 2019). In this process, there is the nucleophilic addition of the free amino groups from proteins, lipids or nucleic acids to the carbonyl groups of monosaccharides. This reaction (known as Maillard reaction) forms a reversible Schiff base adduct that spontaneously undergoes Amadori rearrangement to form the reactive dicarbonyls or ketamines when the attachment becomes irreversible (Vasudevan et al., 2013; Scheijen et al., 2018; Richard et al., 2019) as shown in Figure 1.

These AGEs can be formed endogenously when large amounts of monosaccharides are present or exogenously during food processing (Richard et al., 2019). It has been reported that intake of foods which are high in monosaccharides and/or foods that were exposed to high temperature cooking such as deep-frying, boiling, roasting, baking and grilling increases the total daily intake of AGEs by 25% (Estifanos et al., 2017) thereby increasing the concentration of circulating AGEs when absorbed (Sang and Young, 2018; Richard et al., 2019).

Although AGEs are formed to their elevated levels in the body during hyperglycemia, studies have also shown that AGEs formation may be stimulated even in normoglycemic conditions such as: renal failure, oxidative stress, and inflammatory conditions (Ravichandran et al., 2008).

## Receptor for Ages and Cell Signaling

The formation of AGEs has been shown to activate different signaling pathways which are mediated by different types of cell surface receptors, one of which is the multi-ligand receptor for advanced glycation end products (RAGE) (Christiane et al., 2014). However, other AGEs-receptors as described in Table 2 have also been identified and their expression depends on the type of cell/tissue and the metabolic needs (Ohgami et al., 2001, 2002; Christiane et al., 2014). But for the purpose of this review, we are focusing on RAGE.

**Table 2:** Receptors for advanced glycation end products and their functions.

No	AGE binding protein	Molecular weight	Ligands	Expressions/Cell Types	Functions
1	RAGE	F1 RAGE-43 to 55 kilodaltons (kDa) sRAGE-36 to 50 kDa N-Truncated RAGE-32 to 42 kDa	S100 proteins, HMGB-1, β-amyloid, amyloid fibrils, β-integrin Mac-1, AGEs	Monocytes/macrophage, T-lymphocytes, endothelial cells, mesangial cells, fibroblasts, smooth muscle cells, neuronal cells	F1 RAGE and N-Truncated RAGE (endocytosis, cell signaling); sRAGE (attenuate inflammation and free radicals, decoy receptors for AGEs) (Christiane et al., 2014)
2	AGE-R1 (OST-48)	50 kDa	AGEs, glucose modified proteins	Monocytes/macrophage, T-lymphocytes, endothelial cells, mesangial cells, fibroblasts, smooth muscle cells, neuronal cells	Take up and removes circulating AGEs, Decrease the expression of the prooxidant-p66^shc^ (Cai et al., 2007, 2008; Christiane et al., 2014; Uribarri, 2017)
3	AGE-R2 (80K-H)	59–80 kDa	AGEs	Monocytes/macrophage, T-lymphocytes, endothelial cells, mesangial cells, fibroblasts, smooth muscle cells, neuronal cells	Binding of AGEs, sensor for Ca^2+^, translocates GLUT 4 to the plasma membrane (Hodgkinson et al., 2005)
4	AGE-R3 (galectin-3)	26–32 kDa	AGEs, IgE, CD66, lipopolysaccharides, collagen IV, etc.	Monocytes/macrophage, T-lymphocytes, endothelial cells, mesangial cells, fibroblasts, smooth muscle cells, neuronal cells	Removal of AGEs, inhibition of inflammation, endocytosis of modified low density lipoproteins
5	SR-A (I/II)	50–220 kDa	Low density lipoprotein from human plasma (AcLDL), oxidized low density lipoprotein (OxLDL), AGE, *Escherichia coli*, *Staphylococcus aureus*	Monocytes/macrophage, endothelial and dendritic cells	Take up and degrades modified Low density lipoproteins and some AGE-modified proteins (Christiane et al., 2014)
6	SR-B/CD36	53–88 kDa	AGEs, AcLDL, OxLDL, HDL, low density lipoprotein, very low density lipoprotein, long chain fatty acids, phospholipids, apoptotic cells, *Staphylococcus aureus*	Platelets, endothelial and epithelial cells, adipocytes, B-lymphocytes	Cell adhesion, regulates the transport of fatty acids, Take up and remove circulating AGEs (Christiane et al., 2014)
7	SR-BI	60–76 kDa	AGEs, AcLDL, OxLDL, LDL, HDL, VLDL, phospholipids	Liver, steroidogenesis	Take up and remove AGEs; take up high density lipoproteins (Christiane et al., 2014)
8	SR-E/LOX-1	31–50 kDa	AGEs, oxidized low density, apoptotic cells, activated platelets, *Staphylococcus aureus*, *Escherichia* *coli*	Smooth muscles, endothelial cells, macrophages	Cell signaling, take up and degrade modified oxidized low density lipoproteins (Christiane et al., 2014)
9	FEEL-1/FEEL-2	154–277 kDa	AGEs, AcLDL, secreted protein acidic and rich in cysteine, hyaluronic acid; **Staphylococcus aureus**,**Escherichia coli**	Monocytes/macrophages, endothelial cells	Take up and remove AGEs, hyaluronic acid and AcLDL Christiane et al. (2014)

RAGE is a multiligand, cell-surface receptor, with a molecular weight of 35 kDa, that belongs to the family of immunoglobulins (Srikanth et al., 2011; Valentina et al., 2012) and is composed of 404 amino acids (Parisa and Ali, 2011).

**FIGURE 2:**
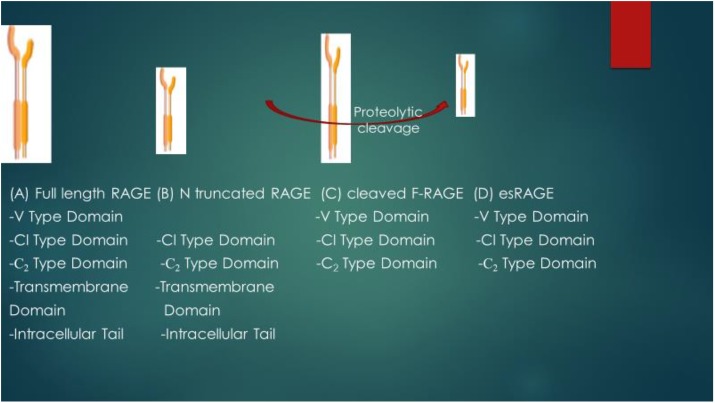
Schematic representation of structural differences of RAGE isoforms. V type domain- 23–116 a.as; Cl type domain: 124–221 a.as; C2 type domain: 227–317 amino acids; Transdomain-343–363 a.as; Intracellular tail: 364-404 a.as. Full length RAGE (F-RAGE) contains V type domain (required for ligand binding), Cl type domain, C_2_ type domain, Transmembrane domain and Intracellular tail. Removal of the V type domain leads to the formation of N truncated RAGE. Cleaved F-RAGE and esRAGE make up the soluble form of RAGE (both lack the intracellular tail and transmembrane domains required for signal transduction) and they may be obtained from proteolytic cleavage of F-RAGE or alternative splicing of mRNA of RAGE.

RAGE is expressed in four main isoforms as shown in Figure 2: the full length RAGE (F-RAGE) and N terminal truncated RAGE which are retained in the plasma membrane, the sRAGE which is present in two isoforms- cleaved full length RAGE or cleaved RAGE (cRAGE) that is produced through proteolytic shedding of the RAGE ectodomain and the COOH-truncated variant, otherwise known as endogenous secretory RAGE (esRAGE) that is formed by alternative splicing of the pre-RNA of RAGE (Parisa and Ali, 2011; Valentina et al., 2012; Miranda et al., 2018). Therefore, cRAGE and esRAGE make up the total sRAGE isoforms (Ryan et al., 2019). While the N-truncated RAGE is incapable of binding with AGEs and has unknown biological function due to lack of V-domain that is required for ligand binding (Koyama et al., 2007), studies have shown that binding of AGE or RAGE ligand leads to the activation of full length RAGE (which responds to proinflammatory conditions), and this increases the expression of RAGE which enhances or exacerbates inflammatory conditions (Lue et al., 2009). This interaction has been shown to worsen or enhance the progression of different disease conditions (Lue et al., 2009).

It has been reported that alternative splicing plays an essential contribution to the production of different isoforms of RAGE as during alternative RNA splicing, exons or introns could be retained or removed in different combinations, leading to the production of different isoforms with their different characteristics (Parisa and Ali, 2011).

sRAGE is the extracellular ligand binding domain of RAGE. It flows in the human plasma and serves as a decoy receptor for RAGE signaling by binding to and sequestering circular plasma AGEs or RAGE ligands, thereby attenuating the signaling of RAGE (Parisa and Ali, 2011). RAGE is reported to be expressed in monocytes, macrophages, microglia, astrocytes, neurons, smooth muscle, and endothelial cells (Parisa and Ali, 2011). Hence, the soluble forms of RAGE (esRAGE and sRAGE) inhibit the interactions between RAGE ligands and full length RAGE, thereby diminishing the activation and expression of RAGE as these isoforms of RAGE contain functional ligand-binding domains but lack cellular signaling domains (Lue et al., 2009). These interactions are summarized in Table 3.

**Table 3:** Interaction between AGEs and RAGE isoforms.

No	RAGE Isoforms	Location of expression	Effect of interaction with AGEs
1	Full length RAGE	Cell surface (Lue et al., 2009; Yaw et al., 2013)	Leads to the activation of RAGE, inflammatory signaling and oxidative stress (Lue et al., 2009; Yaw et al., 2013).
2	N terminal truncated RAGE	Cell surface (Lue et al., 2009; Yaw et al., 2013)	Does not bind to AGEs due to lack of the V-type domain that is essential for ligand binding. Unknown biological function (Koyama et al., 2007; Yaw et al., 2013)
3	esRAGE	Extracellular space (Lue et al., 2009; Yaw et al., 2013)	Binds to AGEs and removes them from circulation. Diminishes AGE-RAGE mediated inflammatory condition and oxidative stress (Lue et al., 2009).
4	sRAGE	Extracellular space (Yaw et al., 2013)	Binds to AGEs and removes them from circulation. Diminishes AGE-RAGE mediated inflammatory condition and oxidative stress (Lue et al., 2009; Yaw et al., 2013)

The mitogen activated protein kinases (MAPKs) such as the extracellular signal-regulated kinase and c-Jun N-terminal kinase (JNK) (also in the family of MAPKs) such as: extracellular signal-regulated kinase (ERK)1/2, and others, were reported to be components of the RAGE signaling pathways (Parisa and Ali, 2011). These MAPKs are induced by cytokines and stressors. Activation of the signaling pathway further activates transcription factors such as: nuclear factor-kappaB (NF-κB) and others. These therefore make MAPKs and NF-κB key components of the RAGE/AGE signaling pathway.

Therefore, AGEs-RAGE interaction activates NF-κB through these MAPKs signaling pathways. Activation of the signaling cascade depends on the interaction between AGEs and RAGE, as sequestering RAGE with either an excess of sRAGE or anti-RAGE antibody prevents cellular activation (Parisa and Ali, 2011). Other ligands (apart from AGEs) that have been found to be recognized by RAGE include: the amyloid β-peptide, DNA binding protein high mobility group box-1 (HMGBl)/amphoterin, and S100/calgranulins (Parisa and Ali, 2011).

When bound to its ligand, RAGE-ligand activates an inflammatory signaling cascade, culminating in the activation of NF-κB and thereafter, transcription of inflammatory cytokines. This has been implicated in the development of oxidative stress, obesity and obesity-mediated diabetes, and atherosclerosis (Chih-Tsueng et al., 2014; Miranda et al., 2018).

**FIGURE 3:**
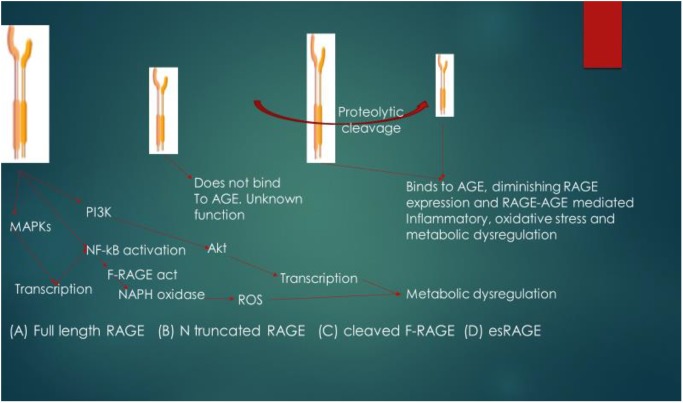
Pathophysiological relevance and signaling functional differences of isoforms of RAGE. The ligand for RAGE binds to and activates cleaved Full length RAGE (F-RAGE) and the signals are transmitted to three pathways (MAPKs, NF-κB, and PI3K), resulting in inflammatory signaling. Activation of NF-κB further promotes RAGE activation (feed-forward) and signaling, leading to the activation of NADPH oxidase and generation of reactive oxygen species (ROS), resulting to metabolic dysregulation. N-truncated RAGE does not bind to AGE due to lack of the V type domain and it has unknown biological function. Binding of the ligand (in this case AGE) to cleaved F-RAGE or es-RAGE diminishes the expression of RAGE, attenuating inflammatory signaling, oxidative stress and metabolic dysregulation. MAPKs, mitogen-activated protein kinase; PI3K, phosphoinositide 3-kinase; NF-κB, nuclear factor-kappa B; Akt, protein kinase B; F-RAGE act, activation of F-RAGE.

esRAGE and sRAGE act as competitive inhibitors of AGE and as such, they decrease the affinity of AGE for other RAGE. The suggested mechanism of action of the soluble forms of RAGE is that AGE interacts with them (esRAGE and sRAGE) in the extracellular environment, thus inhibiting AGE interaction with F-RAGE (Lue et al., 2009), and thereby diminishing the expression of RAGE as these isoforms of RAGE contain functional ligand-binding domains but lack the cellular signaling domains. The pathophysiological relevance and signaling functional differences of isoforms of RAGE are shown in Figure 3. Another signaling pathway that has also been reported to be activated by RAGE-ligand interaction is the Signal transducer and activator of transcription 1 pathway and this pathway has been implicated in RAGE-ligand mediated inflammation (Ravichandran et al., 2008; Juranek et al., 2015).

## Metabolic Effects of Interactions Between Ages and Rage

The AGEs are known to deposit in vessel wall, tissues and endothelium, whereby they alter the structures of extracellular matrix, cell surface receptors, and the functions of intracellular proteins. Interactions between AGEs and their receptors trigger oxidative stress, proinflammatory conditions as well as increased vascular cell adhesion molecule-1 and tumor necrosis factor-alpha expression (Chih-Tsueng et al., 2014; Raffaella et al., 2015). These responses have been reported to be mediated by the activation of Ikkβ/NFkB- nuclear transcriptional elements that are involved in the production of pro- inflammatory cytokines. As these RAGE are present in various organs such as endothelial cell, vascular smooth muscle, skeletal muscle, and kidney, their interactions with AGEs could interfere with the normal functions of these tissues (Simard et al., 2015; Maria et al., 2016), thus aggravating the process of atherosclerosis, intravascular thrombosis, myocardial infarction/vascular dysfunction, and impaired bone formation which increases the risk of osteoporosis (Chih-Tsueng et al., 2014; Raffaella et al., 2015).

Although increased circulating levels of AGEs could potentially be harmful due to their recognition by RAGE that could culminate in metabolic dysregulation, RAGE signaling can be diminished either through proteolytic cleavage to form the cleaved isoform of RAGE (cRAGE) or by alternative splicing that will lead to the production and cellular expulsion via exocytosis of esRAGE (Ryan et al., 2019).

Different signaling pathways triggered by RAGE ligands were reported to indicate the specific cell type, the time course and duration of activation of RAGE but in most of the cases, expression of RAGE is upregulated in disease conditions or metabolic dysfunction (Ravichandran et al., 2008).

RAGE can be constitutively or inducibly expressed in various cells which depends on the type of cell and the stage of development (Yaw et al., 2013). According to Brett et al. (1993) and Yaw et al. (2013), RAGE is highly expressed in a constitutive manner. In addition, there is a lower expression of RAGE in different adult cells such as cardiomyocytes, neurons, neutrophils, monocytes/macrophages, lymphocytes, dendritic, and vascular endothelial cells compared to the embryonic cells (Yaw et al., 2013).

The constitutive expression of RAGE occurs during the developmental stage of the embryo, and its expression in adulthood is regulated suggesting that the expression of RAGE can be induced in conditions of elevated ligands or/and inflammatory mediators (Yaw et al., 2013). On the contrary, tissues such as the skin and lungs have been reported to constitutively express RAGE at high levels throughout one’s lifetime although the biochemical basis for this condition remains to be fully elucidated (Yaw et al., 2013).

## esRage and sRage as Biomarkers for Rage-Mediated Metabolic Effects

Studies have shown that that sRAGE could be a biomarker for RAGE-mediated disease development, especially vascular diseases (Basta, 2008; Parisa and Ali, 2011). This is especially true considering the fact that when they are present in circulation, AGEs could bind to anyone of the isomers of sRAGE, leading to the sequestering and subsequent removal of AGEs, thereby preventing the interaction and activation of AGEs/RAGE signaling. This thus makes the esRAGE and the sRAGE to have a protective effect against the potential harmful effects of AGEs (Kim et al., 2018).

Recent studies have also shown that attenuation of RAGE signaling through an upregulation of sRAGE and esRAGE could be a veritable approach to treat obesity and prevent the development of its comorbidity (Yaw et al., 2013; Davis et al., 2014; Miranda et al., 2017, 2018; Susana et al., 2018).

Whereas esRAGE and sRAGE are known to act as decoy receptors for AGEs by binding to the extracellular ligands for RAGE, preventing the activation of RAGE on the cell surface and protecting the endothelial cells, the exact mechanism of their AGEs chelating action was not previously fully understood.

However, a recent study that made use of a Quantitative Real Time Polymerase Chain Reaction (RT-PCR) and western blot assay provided an insight as they showed that esRAGE abrogates AGEs- mediated apoptosis and inflammation in endothelial cells by downregulating the expressions of the proapoptotic marker-Bax and inflammatory marker NF-κB while upregulating the expression of the antiapoptotic markerBcl-2 (Guomin et al., 2018).

## Targeting of esRage and sRage Signaling as a Beneficial Therapeutic Approach for Obesity and Its Comorbidity

Supporting evidence has suggested that targeting of the upregulation of esRAGE and sRAGE signaling could be a veritable and promising avenue for the treatment of obesity and its comorbidity (D’Adamo et al., 2011). For instance, previous studies showed that the plasma level of sRAGE had a negative correlation with the BMI; and overweight people with higher BMI reportedly had a lower plasma level of sRAGE than their lean counterparts (Norata et al., 2009; Parisa and Ali, 2011).

In a clinical study that was carried out by D’Adamo et al. (2011) examining the levels of esRAGE and sRAGE in obese prepubertal children with or without liver steatosis, the authors reported significantly lower esRAGE and sRAGE levels in obese prepubertal children which were independently related to liver steatosis. Their study further showed that obesity attenuated the levels of sRAGE and esRAGE thereby increasing their risk of developing early markers of cardiovascular disease as had earlier been reported in those with impaired RAGE system (Koyama and Nishizawa, 2010; Brooke et al., 2011).

In an experimental study that was carried out by Brooke et al. (2011) on a mouse model of obesity and on overweight and obese individuals (a randomized clinical trial), they reported that targeted reduction of AGEs improved renal function and inflammatory profile in the obese mice and humans, respectively.

A clinical trial was conducted by Vazzana et al. (2012) to test the hypothesis that changes in esRAGE levels as a result of excess adiposity and oxidative stress may cause platelet activation in obese women, leading to cardiovascular risk. The authors reported low plasma esRAGE level was associated with reduced circulating adiponectin and enhanced synthesis of thromboxane, mediated by increased lipid peroxidation. Their study further showed that obesity may enhance RAGE hyperactivation and subsequent thromboxane-dependent platelet activation, which in turn may exacerbate obesity-related metabolic and vascular diseases.

In another clinical study that was conducted by Tommaso et al. (2012) on obese children, the authors found that the levels of esRAGE and sRAGE were significantly lower in obese children than their non-obese counterpacts.

In a clinical study that determined alterations in sRAGE and esRAGE in normal-weight and obese children who were born either small and large for gestational age and to determine if birth weight, insulin resistance, and obesity represented independent risk factors, the authors reported decreased sRAGE and esRAGE levels in obese children born small and large for gestational age compared to controls (Valentina et al., 2012).

In a later controlled clinical trial that was conducted by Davis et al. (2014) on normal weight, overweight and obese subjects, the authors reported the following measures of adiposity in young adults were inversely correlated with sRAGE level: weight, waist circumference, BMI, and waist-to-height ratio whereas they found a positive correlation between adiponectin and sRAGE levels.

A cross sectional clinical study that was carried out by Chih-Tsueng et al. (2014) to investigate the relationship between sRAGE, obesity, and metabolic syndrome (MetS) in adolescents showed significant associations between sRAGE and BMI, triacylglycerol, Low Density Lipoprotein-cholesterol, and Homeostatic Model Assessment of Insulin Resistance (HOMA-IR) in both male and female adolescents. The authors further reported that sRAGE level was significantly and inversely correlated with increasing number of components of MetS in the male subjects but did not obtain the same result in the female subjects.

A newer clinical study that was carried out by Hagen et al. (2015) on obese subjects that underwent a dietary intervention study for a period of 6 months showed that the serum levels of sRAGE were significantly and inversely associated with BMI and HOMA-IR. The authors further reported that serum levels of sRAGE at baseline were significantly lower in the study subjects with greater reduction of BMI and therefore concluded that sRAGE could be a potential future biomarker to predict weight loss and improvement of insulin resistance in obese subjects who were undergoing a dietary intervention.

In a later clinical study that was carried out by Metin et al. (2016) to determine the association between the serum levels of sRAGE and anthropometric and metabolic parameters in patients with prediabetes and obese controls, the authors reported a negative correlation between sRAGE level and MetS as well as body measurements which was indicative of obesity in the prediabetic state.

In another clinical study that was conducted on normal weight and obese women, Elena et al. (2016) reported significantly lower sRAGE levels in the obese women compared to their normal weight counterparts and the levels of sRAGE in these subjects were correlated inversely with BMI. Their study further showed that low sRAGE levels were reflective of advanced metabolic complications in adult obese women and could be an early marker of cardiometabolic risk.

Another clinical study that was conducted by Mohamed et al. (2016) in obese and non-obese adolescents showed that decreased sRAGE levels were associated with cardiovascular risks in obese adolescents.

In an experimental study that was carried out by Xiong et al. (2017) on mice model of obesity, the authors found significantly elevated AGEs level in obese rats with fatty liver, altered fatty acid metabolism as well as increased TNF-α and IL-6 levels. Their study showed that application of the AGEs inhibitor aminoguanidine positively modulated liver functions and fatty acid metabolism, and also attenuated the TNF-α or IL-6 levels of the obese rats when compared with the non-obese mice. Their study therefore showed that inhibition of AGEs could be a beneficial approach for treating obesity-induced fatty liver disease. Their finding is of significance when considering the importance of sRAGE and esRAGE that function in eliminating or sequestering circulating AGEs.

In another recent study that was carried out by Miranda et al. (2017) on obese subjects, the authors reported that total sRAGE and the isoforms- cRAGE, and esRAGE had a negative correlation with BMI and percent of body fat, with esRAGE having the strongest relationships between both variables. Furthermore, the authors found the circulating sRAGE isoforms to be diminished in obese and impaired glucose tolerant individuals, thus enhancing their risk of developing T2DM.

A similar clinical trial that was conducted by Moushira et al. (2017) on Egyptian obese women found serum levels of sRAGE to be significantly lower in obese cases than controls and correlated inversely with obesity and parameters of MetS. Their study further suggested the potentials of sRAGE as a biomarker for metabolic dysfunction in obese women.

A clinical study that was conducted by Koborová et al. (2017) to determine the levels of circulating sRAGE as a biomarker of the risk of developing MetS and cardiovascular disease in centrally obese women considered metabolically healthy in comparison with the metabolically unhealthy ones, showed that central obesity correlated with low sRAGE levels. Other recent studies also found a significant negative association between measures of obesity (BMI and others) and MetS versus sRAGE and esRAGe levels (Dozio et al., 2017; Koborová et al., 2017).

In a more recent clinical trial that was carried out by Miranda et al. (2018) on obese subjects to determine the effects of weight reduction on sRAGE isoforms, the authors found that esRAGE level increased with improvements in the body composition of the obese subjects and also correlated with markers of adipocyte health, implicating sRAGE as a potential target for the treatment of obesity and cardiovascular diseases.

While obesity can predispose an individual to MetS, T2DM and cardiovascular diseases as earlier stated, it has been reported that not all obese subjects develop MetS (healthy obesity) (Uribarri et al., 2015). However, a clinical study that was carried out by Uribarri et al. (2015) showed that AGEs correlated with the factors that are involved in MetS, such as inflammation and insulin resistance (IR). According to the authors, serum levels of AGEs directly correlated with markers of IR and inflammation (HOMA, leptin, TNFα, and RAGE) and inversely correlated with SIRT1, AGER1, Glyoxalase-I and Adiponectin levels. Their study therefore showed that high serum levels of AGEs may indicate obese individuals at-risk for MetS, T2DM and cardiovascular disease. This clearly shows the promising role of esRAGE and sRAGE as diagnostic markers for obese individuals who are at risk of developing MetS and cardiovascular disease especially when considering their role in eliminating or sequestering circulating AGEs as previously stated.

## Expression of esRage and sRage in Metabolically Healthy Obese Individuals

In a study that was conducted by Gurecka et al. (2016) on the correlation among soluble receptors for AGEs, soluble vascular adhesion protein-1/semicarbazide-sensitive amine oxidase and cardiometabolic risk markers in apparently healthy adolescents, the authors reported a decline in the levels of circulating sRAGE and esRAGE in metabolically healthy obese adolescents.

Similarly, in the later study that was carried out by Koborová et al. (2017) to determine the levels of circulating sRAGE as a biomarker of the risk of developing MetS and cardiovascular disease in centrally obese women considered metabolically healthy in comparison with the metabolically unhealthy ones, the authors reported that central obesity correlated with low sRAGE levels and elevated markers of inflammation irrespective of the presence or absence of cardiometabolic risk factors as previously stated. These reports therefore suggest that decreased expression of esRAGE and sRAGE in metabolically unhealthy and the ‘so called metabolically healthy obesity’ could reveal obese subjects at risk of developing the metabolic syndrome.

The results of the experimental and clinical studies (which are the major strength of this review) that are reported in this review clearly reveal that the soluble isoforms of RAGE act as decoy receptors for RAGE ligands, removing them from circulation, diminishing the expression of RAGE and thereby attenuating inflammatory signaling, oxidative stress and metabolic dysregulation. Hence, targeting of sRAGE and esRAGE signaling holds future promise as a beneficial pharmacotherapeutic approach or could be an important strategy for the treatment of obesity and its comorbidity.

While this review underscores the need for the development of an esRAGE/sRAGE targeted pharmacotherapy as a treatment approach for obesity and its comorbidity, a key thing that must be taken into consideration is the duration and effect of RAGE sequestration or elimination in humans as RAGE has also been reported to play some beneficial roles in human physiology (Parisa and Ali, 2011; Yaw et al., 2013).

In conclusion, this review clearly reveals the potentials of esRAGE and sRAGE as futuristic beneficial pharmacotherapeutic targets for obesity which could be further explored in the search for alternative approach to the treatment of obesity and its comorbidity.

## Author Contributions

CE, MM, NN, NO, OL, and BY conceived the manuscript. CE, MM, and NN reviewed the literature, wrote the first draft of the manuscript, and also edited the manuscript. All authors approved the final draft of the manuscript.

## Conflict of Interest Statement

The authors declare that the research was conducted in the absence of any commercial or financial relationships that could be construed as a potential conflict of interest.

## Footnotes

1 https://www.who.int/news-room/fact-sheets/detail/obesity-and-overweight

2 https://www.who.int/gho/ncd/risk_factors/overweight_text/en/

3 https://www.who.int/news-room/fact-sheets/detail/obesity-and-overweight
