# Corrigendum: Obesity and Comorbidity: Could Simultaneous Targeting of esRAGE and sRAGE Be the Panacea?

**DOI:** 10.3389/fphys.2019.01017

**Published:** 2019-08-07

**Authors:** Chinedum Eleazu, Norsuhana Omar, Oon Zhi Lim, Boon Seng Yeoh, Nik Hazlina Nik Hussain, Mahaneem Mohamed

**Affiliations:** ^1^Department of Physiology, School of Medical Sciences, Universiti Sains Malaysia, Kubang Kerian, Malaysia; ^2^Department of Chemistry/Biochemistry/Molecular Biology, Alex Ekwueme Federal University, Ndufu-Alike, Ikwo, Nigeria; ^3^Women's Health Development Unit, Universiti Sains Malaysia, Kubang Kerian, Malaysia

**Keywords:** obesity, nutrition, metabolic dysregulation, receptor for advanced glycation end products, metabolic syndrome

In the original article, the reference for “Jaime et al. (2015)” was incorrectly written as “Jaime, U., Weijing, C., Mark, W., Elizabeth, T., Laurie, G., Renata, P., et al. (2015). Elevated serum advanced glycationendproducts in obese indicate risk for the metabolic syndrome: a link between Healthy and Unhealthy Obesity? *J. Clin. Endocrinol. Metab*. 100, 1957–1966. doi: 10.1210/jc.2014-3925.” It should be “Uribarri, J., Cai, W., Woodward, M., Tripp, E., Goldberg, L., Pyzik, R., et al. (2015). Elevated serum advanced glycation endproducts in obese indicate risk for the metabolic syndrome: a link between healthy and unhealthy obesity? *J. Clin. Endocrinol. Metab*. 100, 1957–1966. doi: 10.1210/jc.2014-3925.”

The reference has been updated and the citation has been corrected to Uribarri et al. (2015) in the section ***Targeting of esRage and sRage Signaling as a Beneficial*** ***Therapeutic Approach for Obesity and Its Comorbidity***, paragraph 18 and should read:

“While obesity can predispose an individual to MetS, T2DM and cardiovascular diseases as earlier stated, it has been reported that not all obese subjects develop MetS (healthy obesity) (Uribarri et al., 2015). However, a clinical study that was carried out by Uribarri et al. (2015) showed that AGEs correlated with the factors that are involved in MetS, such as inflammation and insulin resistance (IR). According to the authors, serum levels of AGEs directly correlated with markers of IR and inflammation (HOMA, leptin, TNFα, and RAGE) and inversely correlated with SIRT1, AGER1, Glyoxalase-I and Adiponectin levels. Their study therefore showed that high serum levels of AGEs may indicate obese individuals at-risk for MetS, T2DM and cardiovascular disease. This clearly shows the promising role of esRAGE and sRAGE as diagnostic markers for obese individuals who are at risk of developing MetS and cardiovascular disease especially when considering their role in eliminating or sequestering circulating AGEs as previously stated.”

The reference for “Judyta et al. (2015)” was also incorrectly written as “Judyta, K. J., Gurdip, K. D., Joanna, W., David, L., Julia, K., and Ann, M.S. (2015). Receptor for advanced glycation end products and its inflammatory ligands are upregulated in amyotrophic lateral sclerosis. *Front. Cell. Neurosci*. 9:485. doi: 10.10.3389/fncel.2015.00485.” It should be “Juranek, J. K., Daffu, G. K., Wojtkiewicz, J., Lacomis, D., Kofler, J., and Schmidt, A.M. (2015). Receptor for advanced glycation end products and its inflammatory ligands are upregulated in amyotrophic lateral sclerosis. *Front. Cell. Neurosci*. 9:485. doi: 10.3389/fncel.2015.00485.”

The reference has been updated and the citation has been corrected to Juranek et al. (2015) in the section ***Receptor for Ages and Cell Signaling***, paragraph nine and should read:

“esRAGE and sRAGE act as competitive inhibitors of AGE and as such, they decrease the affinity of AGE for other RAGE. The suggested mechanism of action of the soluble forms of RAGE is that AGE interacts with them (esRAGE and sRAGE) in the extracellular environment, thus inhibiting AGE interaction with F-RAGE (Lue et al., 2009), and thereby diminishing the expression of RAGE as these isoforms of RAGE contain functional ligand-binding domains but lack the cellular signaling domains. The pathophysiological relevance and signaling functional differences of isoforms of RAGE are shown in Figure 3. Another signaling pathway that has also been reported to be activated by RAGE-ligand interaction is the Signal transducer and activator of transcription 1 pathway and this pathway has been implicated in RAGE-ligand mediated inflammation (Ravichandran et al., 2008; Juranek et al., 2015).”

The reference for “Kathleen et al. (2014)” was also incorrectly written as “Kathleen, E. D., Chandan, P., Parakat, V., Shanil, J., and Victorine, I. (2014). Serum soluble receptor for advanced glycation end products correlates inversely with measures of adiposity in young adults. *Nutr. Res*. 34, 478–485. doi: 10.1016/j.nutres.2014.04.012.” It should be “Davis, K. E., Prasad, C., Vijayagopal, P., Juma, S., and Imrhan V. (2014). Serum soluble receptor for advanced glycation end products correlates inversely with measures of adiposity in young adults. *Nutr. Res*. 34, 478–485. doi: 10.1016/j.nutres.2014.04.012.”

The reference has been updated and the citation has been corrected to Davis et al. (2014) in the section ***esRage and sRage as Biomarkers for Rage-Mediated Metabolic Effects***, paragraph two:

“Recent studies have also shown that attenuation of RAGE signaling through an upregulation of sRAGE and esRAGE could be a veritable approach to treat obesity and prevent the development of its comorbidity (Yaw et al., 2013; Davis et al., 2014; Miranda et al., 2017, 2018; Susana et al., 2018).”

An additional correction has been made to the section ***Targeting of esRage and sRage*** ***Signaling as a Beneficial Therapeutic Approach for Obesity and Its Comorbidity***, paragraph seven and should read:

“In a later controlled clinical trial that was conducted by Davis et al. (2014) on normal weight, overweight and obese subjects, the authors reported the following measures of adiposity in young adults were inversely correlated with sRAGE level: weight, waist circumference, BMI, and waist-to-height ratio whereas they found a positive correlation between adiponectin and sRAGE levels.”

Additionally, in the original article, “Koborova et al. (2017)” and “Ivana et al. (2017)” were cited as two different citations however they both cite the same publication. The correct citation is: “Koborova, I., Gurecka, R., Csongova, M., Volkovova, K., Szöko, E., Tábi, T., et al. (2017). Association between metabolically healthy central obesity in women and levels of soluble receptor for advanced glycation end products, soluble vascular adhesion protein-1, and the activity of semicarbazide-sensitive amine oxidase. *Croat. Med. J*. 58, 106–116. doi: 10.10.3325/cmj.2017.58.106.”

The incorrect reference has been removed and the citation has now been corrected to Koborova et al. (2017) in the section ***Targeting of esRage and sRage Signaling as a*** ***Beneficial Therapeutic Approach for Obesity and Its Comorbidity***, paragraph 16:

“A clinical study that was conducted by Koborova et al. (2017) to determine the levels of circulating sRAGE as a biomarker of the risk of developing MetS and cardiovascular disease in centrally obese women considered metabolically healthy in comparison with the metabolically unhealthy ones, showed that central obesity correlated with low sRAGE levels. Other recent studies also found a significant negative association between measures of obesity (BMI and others) and MetS versus sRAGE and esRAGe levels (Dozio et al., 2017; Koborova et al., 2017).”

An additional correction has been made in the section ***Expression of esRage and*** ***sRage in Metabolically Healthy Obese Individuals***, paragraph two and should read:

“Similarly, in the later study that was carried out by Koborova et al. (2017) to determine the levels of circulating sRAGE as a biomarker of the risk of developing MetS and cardiovascular disease in centrally obese women considered metabolically healthy in comparison with the metabolically unhealthy ones, the authors reported that central obesity correlated with low sRAGE levels and elevated markers of inflammation irrespective of the presence or absence of cardiometabolic risk factors as previously stated. These reports therefore suggest that decreased expression of esRAGE and sRAGE in metabolically unhealthy and the ‘so called metabolically healthy obesity' could reveal obese subjects at risk of developing the metabolic syndrome.”

Lastly, “Natale et al. (2012)” and “Vazzana et al. (2012)” were cited as two different citations however they both cite the same publication. The correct citation is: “Vazzana, N., Guagnano, M. T., Cuccurullo, C., Ferrante, E., Stefano Lattanzio, S., Liani, R., et al. (2012). Endogenous secretory RAGE in obese women: association with platelet activation and oxidative stress. *J. Clin. Endocrinol. Metab*. 97, E1726–E1730. doi: 10.1210/jc.2012-1473.”

The incorrect reference has been removed and the citation has now been corrected in the section ***Targeting of esRage and sRage Signaling as a Beneficial Therapeutic Approach for*** ***Obesity and Its Comorbidity***, paragraph four:

“A clinical trial was conducted by Vazzana et al. (2012) to test the hypothesis that changes in esRAGE levels as a result of excess adiposity and oxidative stress may cause platelet activation in obese women, leading to cardiovascular risk. The authors reported low plasma esRAGE level was associated with reduced circulating adiponectin and enhanced synthesis of thromboxane, mediated by increased lipid peroxidation. Their study further showed that obesity may enhance RAGE hyperactivation and subsequent thromboxane-dependent platelet activation, which in turn may exacerbate obesity-related metabolic and vascular diseases.”

The reference for “Uribarri, J. (2017)” was incorrectly written with the first names as “Jaime, U. (2017). *Dietary AGEs and Their Role in Health and Disease*. Available at: https://books.google.com.my/books?isbn=1351646354 (accessed March 10, 2019).” It should be “Uribarri, J. (2017). *Dietary Advanced Glycation End Products and Their Role in Health and Disease*. Available at: https://books.google.com.my/books?isbn=1351646354 (accessed March 10, 2019).”

The reference has been updated and the citation has been corrected in Table 2. Uribarri (2017) was incorrectly cited with the first name as: Jaime (2017). The correct citation is: Uribarri (2017).

The authors apologize for these errors and state that this does not change the scientific conclusions of the article in any way. The original article has been updated.
